# Effect of high-quality nursing intervention on anxiety and depression in patients with chronic heart failure companied malnutrition: A protocol for systematic review and meta-analysis

**DOI:** 10.1097/MD.0000000000020261

**Published:** 2020-05-29

**Authors:** Xiao-mei Yang, Qiu-mei Li, Qing-ning Gao

**Affiliations:** a Department of Clinical Nutrition; b Department of Cardiology, Affiliated Hospital of Jilin Medical University, Jilin, 132013, China.

**Keywords:** anxiety, chronic heart failure, depression, effect, high-quality nursing intervention, malnutrition

## Abstract

**Background:**

This study will assess the effect of high-quality nursing intervention (HQNI) on anxiety and depression in patients with chronic heart failure companied malnutrition (CHFM).

**Methods:**

We will retrieve electronic databases from the respective dates to February 29, 2020 without language and publication status restrictions: Cochrane Library, Web of Science, MEDLINE, EMBASE, Scopus, Chinese Biomedical Literature Database, and China National Knowledge Infrastructure. All potential randomized controlled trials (RCTs), which examined the effect of HQNI on anxiety and depression in patients with CHFM will be included. Two team members will separately perform article retrieval, duplicates excluding, scanning, data collection, and study quality assessment. In addition, this study will carry out data analysis by RevMan 5.3 software.

**Results:**

This study will provide high-quality synthesis and/or descriptive analysis of the latest evidence to assess the effect of HQNI on anxiety and depression in patients with CHFM.

**Conclusion:**

The findings of this study will exert evidence to judge whether or not HQNI is effective on anxiety and depression in patients with CHFM.

**Registration number:**

INPLASY202040069.

## 1 Introduction

Chronic heart failure (CHF) is a serious and complex cardiovascular disease with high mortality.^[1–4]^ The results of epidemiological studies have found that the prevalence of CHF is about 1% to 2% in general population.^[5,6]^ It is characterized by the structural and functional disturbances of heart that affects its ability to pump an adequate blood and oxygen to supply tissues.^[7–11]^ Moreover, most patients with CHF often accompany malnutrition.^[12–15]^ Additionally, patients with chronic heart failure companied malnutrition (CHFM) experience anxiety and depression.^[16,17]^ Fortunately, a variety of studies have reported that high-quality nursing intervention (HQNI) can effectively manage the anxiety and depression in patients with CHFM.^[18–24]^ However, no systematic review has addressed the effect of HQNI for the management of anxiety and depression in patients with CHFM. Therefore, this study will evaluate the effect of HQNI for anxiety and depression in patients with CHFM.

## 2 Methods

### 2.1 Study registration

This study was funded and registered on INPLASY202040069. It was organized based on the guideline of Preferred Reporting Items for Systematic Reviews and Meta-Analysis (PRISMA) Protocol statement.^[25]^

### 2.2 Criteria for included studies

#### 2.2.1 Study types

This proposed study will include randomized controlled trials (RCTs) that examined the effect of HQNI on anxiety and depression in patients with CHFM. We will exclude other studies, such as review, comments, non-clinical trials, and uncontrolled studies.

#### 2.2.2 Participants

All CHFM participants who were diagnosed as anxiety and depression will be included in this study, in spite of their country, race, gender, age, and severity of CHFM.

#### 2.2.3 Interventions

In the intervention group, all patients received HQNI for the management of their anxiety and depression.

In the control group, all patients underwent any treatments with no restrictions, except any forms of HQNI.

### 2.3 Outcomes

#### 2.3.1 Primary outcomes

Depression (as measured by any scale reported in the trial, such as Geriatric Depression Scale);
Anxiety (as measured by any tool reported in the trial, such as Beck Anxiety Inventory).

#### 2.3.2 Secondary outcomes

Nutritional status (as assessed by any scale reported in the trial, such as The Malnutrition Universal Screening Tool);
All-cause mortality;
Urine output;
Change in serum sodium;
Quality of life (as identified by any indexes reported in the trial, such as Physical Quality of Life Index); and
Adverse events.

### 2.4 Strategy of literature searches

The following electronic databases will be sought from the respective dates to February 29, 2020 without language and publication status restrictions: Cochrane Library, Web of Science, MEDLINE, EMBASE, Scopus, Chinese Biomedical Literature Database, and China National Knowledge Infrastructure. All potential RCTs that explored the effect of HQNI on anxiety and depression in patients with CHFM will be included. Exemplary search strategy for Cochrane Library is created (Table 1). We will modify similar search strategies for other electronic databases.

**Table 1:**
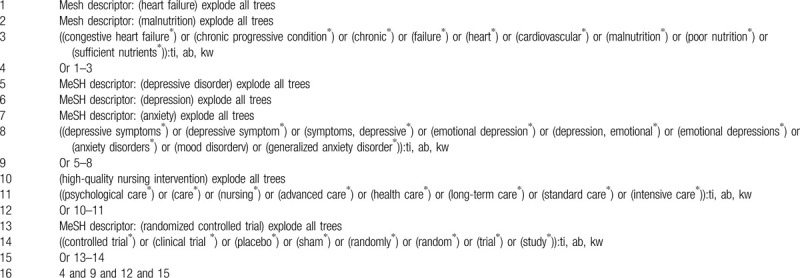
Search strategy of Cochrane Library.

In addition, we will also search other literature sources, such as Google Scholar, conference proceedings, and reference lists of related reviews.

### 2.5 Data collection

#### 2.5.1 Study selection

**Figure 1:**
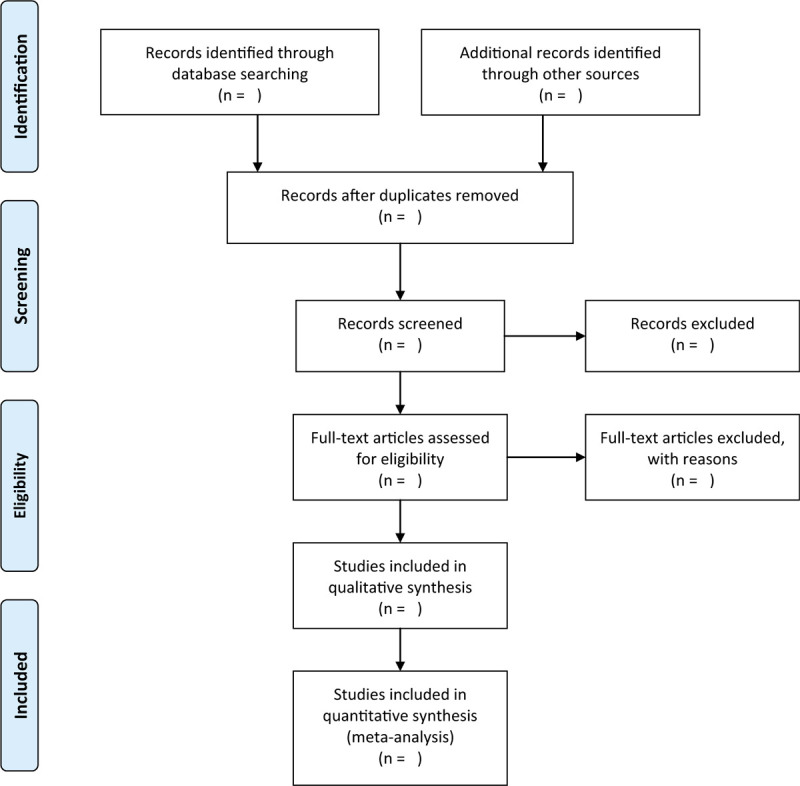
Flowchart of study selection.

Two team members will separately identify searched studies by scanning their titles and abstracts, and all unrelated and repetitive studies will be eliminated. Then, all potential trials will be carefully read against all eligibility criteria after obtaining full papers of potential studies. All selecting operation will be rendered in a PRISMA flowchart (Fig. 1). Any divergences will be figured out by a third team member through discussion.

#### 2.5.2 Data collection

Two team members will separately collect data from included articles using a predefined data extraction form. Any differences will be solved through discussion with a third team member. Collected information is study characteristics (such as title, first author, year of publication, etc), participant characteristics (such as age, gender, duration, and severity of CHFM, anxiety and depression, etc), sample size, study methods, study setting, details of interventions and comparators, outcomes, results, follow-up information, safety, and conflict of interest.

#### 2.5.3 Dealing with missing data

Any unclear or insufficient data will be obtained from original trial authors by email or phone. If that kind of data is not achievable, we will perform data analysis using intention-to-treat analysis.

### 2.6 Study quality assessment for included studies

Cochrane Collaboration Tool will be used to appraise study quality by 2 team members separately. If any disagreements occur between both of them, we will invite a third team member to solve them through discussion, and a consensus will be reached after discussion.

### 2.7 Statistical analysis

We will utilize ReMan 5.3 software to pool the data and to perform data analysis and a meta-analysis if possible. Treatment effect of continuous data will be estimated as weighted mean difference or standardized mean difference and 95% confidence intervals (CIs), and that of dichotomous data will be estimated as risk ratio and 95% CIs. *P* < .05 is considered as having statistically significance.

We will examine statistical heterogeneity by *I*^2^ test. *I*^2^ ≤ 50% indicates a minor heterogeneity, and a fixed-effects model will be applied to pool the data. *I*^2^ > 50% suggests significant heterogeneity, and a random-effects model will be carried to synthesize the data. When there is homogeneity, we will conduct a meta-analysis if sufficient data is collected from eligible trials. Otherwise, we will perform a subgroup analysis to detect possible reasons of significant heterogeneity. If there is still substantial heterogeneity after subgroup analysis, we will not carry out a meta-analysis.

### 2.8 Additional analysis

Subgroup analysis will be examined based on the variations in study and patient characteristics, and different types of treatments, controls, and outcome measurements.

Sensitivity analysis will also be performed to test the robustness of study findings by taking away trials with high risk of bias.

A funnel plot^[26]^ and Egger's regression test^[27]^ will be examined to identify reporting bias if at least 10 studies are included.

### 2.9 Ethics and dissemination

This study will only collect data from previous published studies, thus, no ethic approval is required. The findings of this study will be published on a peer-reviewed journal.

## 3 Discussion

CHFM is a very severe cardiovascular disease that leads to high mortality and morbidity. Moreover, most patients with CHFM also accompany anxiety and depression. Fortunately, previous studies have reported that HQNI is effective on anxiety and depression in patients with CHFM. However, no systematic review has investigated the effect of HQNI for the management of depression and anxiety in patients with CHFM. This study will systematically assess the effect of HQNI for anxiety and depression in patients with CHFM. Its findings will supply rigorous summary evidence and will inform our understanding of HQNI for anxiety and depression in patients with CHFM across all eligible studies.

## Author contributions

**Conceptualization:** Xiao-mei Yang, Qing-ning Gao.

**Data curation:** Xiao-mei Yang, Qiu-mei Li, Qing-ning Gao.

**Formal analysis:** Xiao-mei Yang, Qiu-mei Li.

**Funding acquisition:** Qing-ning Gao.

**Investigation:** Qing-ning Gao.

**Methodology:** Xiao-mei Yang, Qiu-mei Li.

**Project administration:** Qing-ning Gao.

**Resources:** Xiao-mei Yang, Qiu-mei Li.

**Software:** Xiao-mei Yang, Qiu-mei Li.

**Supervision:** Qing-ning Gao.

**Validation:** Xiao-mei Yang, Qiu-mei Li, Qing-ning Gao.

**Visualization:** Xiao-mei Yang, Qing-ning Gao.

**Writing – original draft:** Xiao-mei Yang, Qiu-mei Li, Qing-ning Gao.

**Writing – review & editing:** Xiao-mei Yang, Qiu-mei Li, Qing-ning Gao.
